# Evaluation of quinclorac toxicity and alleviation by salicylic acid in rice seedlings using ground-based visible/near-infrared hyperspectral imaging

**DOI:** 10.1186/s13007-020-00576-7

**Published:** 2020-03-05

**Authors:** Jian Wang, Chu Zhang, Ying Shi, Meijuan Long, Faisal Islam, Chong Yang, Su Yang, Yong He, Weijun Zhou

**Affiliations:** 1 Institute of Crop Science, Ministry of Agriculture and Rural Affairs Key Laboratory of Spectroscopy Sensing, Zhejiang University, Hangzhou, 310058 China; 2 UWA School of Agriculture and Environment and The UWA Institute of Agriculture, Faculty of Science, The University of Western Australia, Crawley, WA 6009 Australia; 3 College of Biosystems Engineering and Food Science, Ministry of Agriculture and Rural Affairs Key Laboratory of Spectroscopy Sensing, Zhejiang University, Hangzhou, 310058 China; 4 Bioengineering Research Laboratory, Guangdong Bioengineering Institute (Guangzhou Sugarcane Industry Research Institute), Guangzhou, 510316 China; 5 College of Life Sciences, China Jiliang University, Hangzhou, 310018 China

**Keywords:** Rice, Quinclorac, Salicylic acid, Antioxidant, Support vector machine, Hyperspectral imaging

## Abstract

**Background:**

To investigate potential effects of herbicide phytotoxic on crops, a major challenge is a lack of non-destructive and rapid methods to detect plant growth that could allow characterization of herbicide-resistant plants. In such a case, hyperspectral imaging can quickly obtain the spectrum for each pixel in the image and monitor status of plants harmlessly.

**Method:**

Hyperspectral imaging covering the spectral range of 380–1030 nm was investigated to determine the herbicide toxicity in rice cultivars. Two rice cultivars, Xiushui 134 and Zhejing 88, were respectively treated with quinclorac alone and plus salicylic acid (SA) pre-treatment. After ten days of treatments, we collected hyperspectral images and physiological parameters to analyze the differences. The score images obtained were used to explore the differences among samples under diverse treatments by conducting principal component analysis on hyperspectral images. To get useful information from original data, feature extraction was also conducted by principal component analysis. In order to classify samples under diverse treatments, full-spectra-based support vector classification (SVC) models and extracted-feature-based SVC models were established. The prediction maps of samples under different treatments were constructed by applying the SVC models using extracted features on hyperspectral images, which provided direct visual information of rice growth status under herbicide stress. The physiological analysis with the changes of stress-responsive enzymes confirmed the differences of samples under different treatments.

**Results:**

The physiological analysis showed that SA alleviated the quinclorac toxicity by stimulating enzymatic activity and reducing the levels of reactive oxygen species. The score images indicated there were spectral differences among the samples under different treatments. Full-spectra-based SVC models and extracted-feature-based SVC models obtained good results for the aboveground parts, with classification accuracy over 80% in training, validation and prediction set. The SVC models for Zhejing 88 presented better results than those for Xiushui 134, revealing the different herbicide tolerance between rice cultivars.

**Conclusion:**

We develop a reliable and effective model using hyperspectral imaging technique which enables the evaluation and visualization of herbicide toxicity for rice. The reflectance spectra variations of rice could reveal the stress status of herbicide toxicity in rice along with the physiological parameters. The visualization of the herbicide toxicity in rice would help to provide the intuitive vision of herbicide toxicity in rice. A monitoring system for detecting herbicide toxicity and its alleviation by SA will benefit from the remarkable success of SVC models and distribution maps.

## Background

Rice (*Oryza sativa* L.) is one of the primary income-generating cereal crops for farmers, with a net approximately 481 dollars per hectare in most of southeast Asia like the Philippines, and is a staple food for more than half of the world’s population depending on rice for more than 20% of their daily calories [1]. However, rice production is heavily constrained by different biotic and abiotic factors, such as drought, high temperature, diseases, heavy metals, herbicides, etc. [2]. Weeds are considered to be as one of the most important biotic factors in rice fields [3]. Researchers have found that if enough hand weeding is done at the optimal times, crop production will not be decreased by weed competition [4]. In fact, paddy fields are scarcely ever weeded by hand since weeding is tedious and time consuming. The increasing cost of labor and their shortage make hand weeding impossible [5]. To ensure rice yields, farmers usually use herbicides to control weeds, because chemical control is cheaper, more reliable, and less labor-intensive and time-consuming compared to manual weeding [6]. The quinolinecarboxylic acid quinclorac (3,7-dichloro-8-quinolinecarboxylic acid), belonging to a new class of highly selective auxin herbicides, has been used as pre- and post-application in transplanted and directly seeded rice [7]. However, excessive herbicide usage can cause damage to crops at early stages [8]. Since 1990, the herbicide quinclorac, due to its high efficiency, has been widely applied in China to control the most noxious weed, barnyard grass in rice fields [9, 10]. In our previous study, quinclorac can cause serious herbicide toxicity symptoms in rice [7]. Thus, how to alleviate the damages caused by herbicides is a critical issue for herbicide damage control.

Nowadays, various approaches, for example, breeding for herbicide-resistant crops, have been utilized to alleviate the toxic issues. Exogenous hormone application is an effective, economical, environment friendly and safe method for crops to alleviate herbicide toxicity [11, 12]. For example, Ananieva et al. [13] found that salicylic acid (SA) treatment decreased the effects of herbicide paraquat on photosynthesis. Application of exogenous jasmonic acid enhanced herbicide tolerance in tobacco (*Nicotiana tabacum*) exposed to imazapic by reducing herbicidal residue effect and up-regulating related stress-responsive enzymes [14]. Gibberellic acid has been demonstrated to protect *Zea mays* from metolachlor toxicity [15]. SA is a plant endogenous hormone that regulates several biochemical processes and plays a crucial role in plant defense systems [16]. Researchers have found that the exogenous application of SA impacts various physiological processes such as photosynthesis, antioxidant capacity, transpiration rate, stomatal closure, membrane permeability and lignin deposition, and it improves drought, chilling and herbicide tolerance [7, 13, 17–19]. Furthermore, according to Grossmann and Kwiatkowski [20] and Wang et al*.* [7], SA alleviates quinclorac toxicity by increasing antioxidant enzyme activities and by enhancing detoxification ability in rice seedlings.

It is quite hard to monitor herbicide’s phytotoxic symptoms of rice in the field. Because sometimes when plants are emerging with spots, chlorosis, abnormality, wilting or growth retardation, it is difficult to determine the reasons that are provoked by herbicide toxicity, diseases, viruses or just nutrition deficiency. Traditionally, farmers can only use their eyes to distinguish whether the crops are under abiotic or biotic stress. Qualitative investigation depends on herbicide application methods, environments, crop harmful symptoms, but these results lack specific data or relevant indicators to support and contain subjective consciousness without a comparatively-consolidated criterion. Quantitative analysis is much harder to discriminate whether plant is under higher herbicide stress. In our previous studies [7], determination of physiological parameters has been utilized to evaluate the plant physiological changes under herbicide stress and its recovery by SA. In fact, it is too late when phytotoxic symptoms become evident, the damage is irreversible and the components of herbicide may have been already degraded in plants. The above-mentioned methods require complex operations and large reagent consumption and are time consuming, which cannot be used for rapid and large-scale detection of herbicide toxicity/crop damage. Therefore, rapid and sensitive techniques are required for measuring herbicide toxicity in rice plants, allowing prompt reactions and remediation to ensure food production and safety.

Multiple herbicide injury identification methods have been evaluated on crops. Obviously, visual examination provides the most direct way to assess occurrence and extent of herbicide injury. However, this approach is possible when injury is apparent to the naked eyes. Other methods include the measurement of physical factors such as plant height [21], chlorophyll [22], some growth-related compounds or enzymes [23]. All of mentioned methods are tedious and lagging in early detection of herbicide injury. Therefore, alternative approaches for early detection of herbicide damage are imperative.

In the herbicide metabolism process, it will influence many physiological processes which include a reduction in chlorophyll content, and decrease in photosynthetic rate, amino acid synthesis and so on [24]. These changes are detectable with plant reflectance measurements before herbicide injury becoming noticeable. It is possible to evaluate herbicide stresses by using spectral reflectance measurements. Non-imaging visible and near-infrared reflectance (VNIR) spectroscopy (400–2500 nm) is one of the most promising techniques based on the absorption of radiation in the visible and near-infrared region of the electromagnetic spectrum for crop analysis [25]. Although near-infrared spectra (NIR) analysis depends on the quality of model established by species, growth stage, available spectral range and some other factors [26], the main advantages of the NIR methods are low costs, quick and accurate responses, non-destructive analysis, and no need for or minimum sample preparation or manipulation with hazardous chemicals/solvents [27]. Previously, rice crop VNIR has been utilized for the detection of chlorophyll, nitrogen content, moisture content, starch quality, protein activity and amino acid content [28–32]. Wu et al. [33] and Sánchez et al. [34] used NIR technology to determine herbicide or pesticide levels in vegetables and food. However, there were few reports on herbicide quinclorac toxicity detection in rice plants using NIR. Non-imaging VNIR can acquire spectra from small sample region of the sample, which lacks the acquisition of spatial information.

As an extension of both spectroscopic and imaging techniques, hyperspectral imaging (HSI) has become an emerging platform. HSI integrates two classical optical sensing technologies of computer imaging vision and spectroscopy to obtain both spatial and spectral information from an object [35]. The generated spatial map of spectral variation is capable of determining simultaneously the inherent physical and chemical properties of samples as well as their spatial distribution [36]. For a hyperspectral image, each pixel contains a spectrum at the spectral range of the HSI system. Therefore, it is used to visualize the distribution of quality parameters of different samples, which provides direct visual information of the entire samples [37]. HSI has been used for remote sensing [38] and ground-based sensing in agriculture [39–41]. As a fast and non-destructive method, HSI has been widely applied in agricultural fields for mapping crop seeds, nutrition and diseases [39–41].

Moreover, the spectral range from 400 nm to around 1000 nm is widely used in monitoring plant growth for VNIR and HSI [42, 43]. Researchers have found that the spectral range (400–700 nm) was mainly influenced by pigments and the spectral range (700–1000 nm) was mainly influenced by leaf or canopy structure [44–48]. What is more, vegetation indices (including the red edge parameter) which are widely used to monitor plant growth, are derived from the spectral range between 400 and 1000 nm [47, 49, 50]. Researchers have used the spectra in this range to detect plant stresses, such as drought, water deficiency, salinity and disease [41, 51–53]. Moreover, spectra in this range were also applied to study the changes of plant growth under herbicide or pesticide stress [54, 55].

**Fig. 1:**
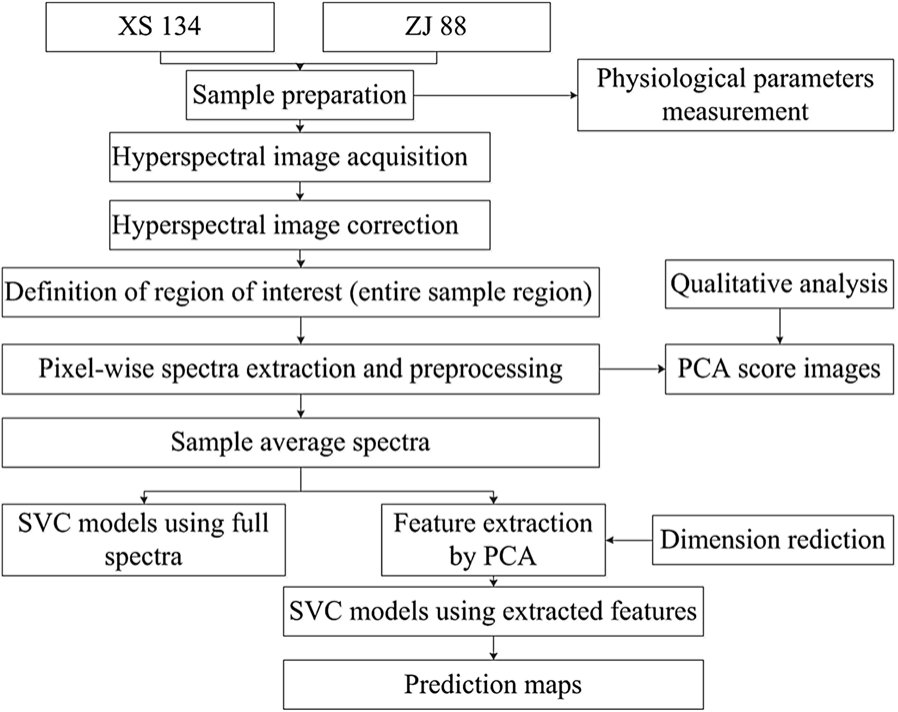
The flowchart of the study design

The prime aim of the present study was to evaluate the feasibility of using ground-based HSI for the separability of quinclorac toxicity and the alleviation function of SA in rice seedlings. The specific objectives were: (1) comparison of spectral variations of leaves under normal and quinclorac stress, and stress alleviation by SA; (2) establishment of detection models and visualization of leaves under different treatments; and (3) investigation of impact of rice varieties Xiushui 134 (XS 134) and Zhejing 88 (ZJ 88) on detection performance. The general workflow of this study is shown in Fig. 1.

## Results

### Analysis of physiological parameters

**Fig. 2:**
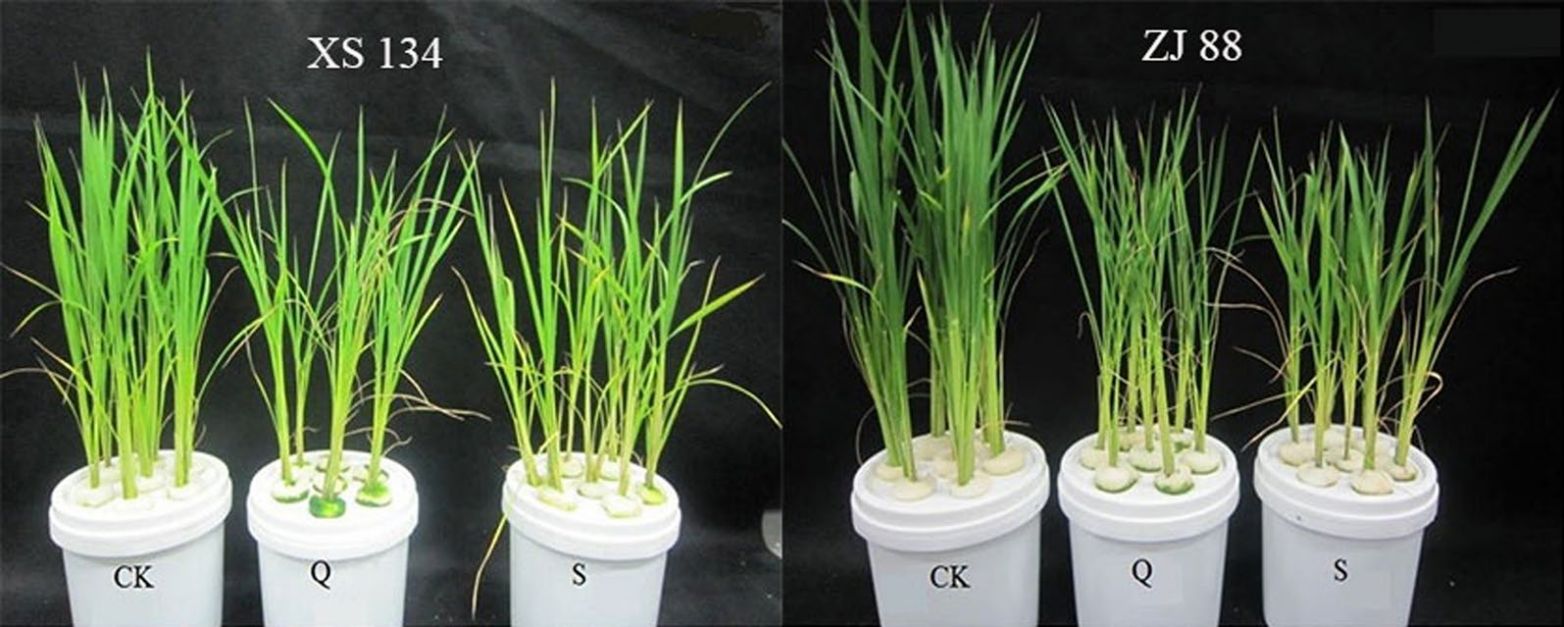
The growing photos of two rice cultivars under different treatments. CK: control; Q: 0.25 g/L quinclorac; S: 10 mg/L SA pretreatment followed by 0.25 g/L quinclorac

The experimental design is shown in Table 1. It is clear that under quinclorac stress, rice seedlings were inhibited with leaf rolling, especially in cv. ZJ 88. Our previous study has demonstrated that SA pre-treatment can alleviate growth damage in both two cultivars (cv. XS 134 and cv. ZJ 88). However, we can’t distinguish the significant difference between quinclorac treated plant (Q) group and SA pre-treatment plant (S) group. Even in XS 134, there was no big difference among three different treatments since XS 134 is more resistant to quinclorac than ZJ 88 (Fig. 2).

**Table 1:** Experimental design

Treatment	Quinclorac (g/L)	Salicylic acid (mg/L)
Control (CK)	0	0
Quinclorac alone (Q)	0.25	0
Salicylic acid pretreatment and quinclorac (S)	0.25	10

**Fig. 3:**
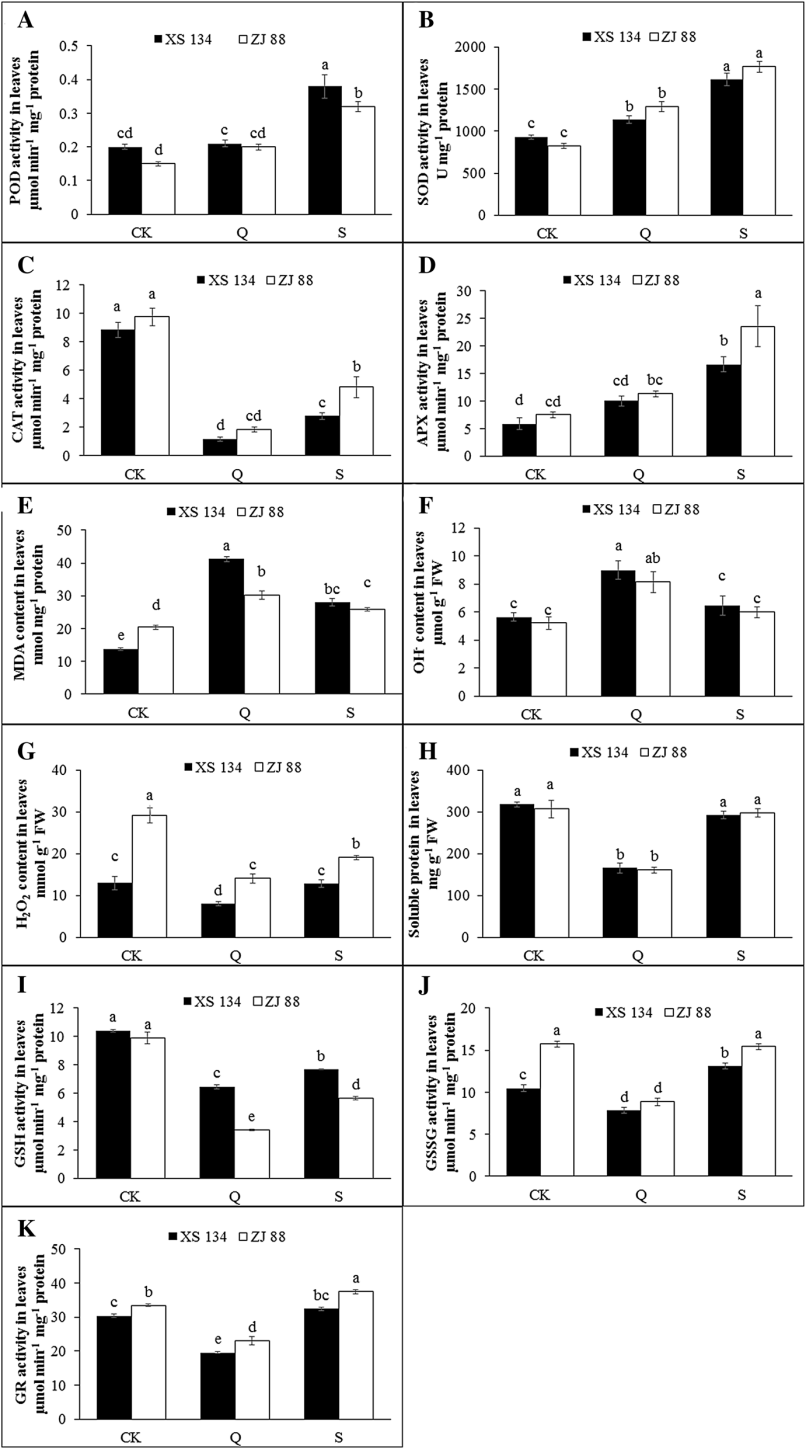
Effects of different treatments on the activities of POD (**A**), SOD (**B**), CAT (**C**), APX (**D**), MDA (**e**), H_2_O_2_ (**F**), OH^−^ (**G**), soluble protein (**H**), GSH (**I**), GSSG (**J**), GR (**K**) in leaves of two rice cultivars respectively. Data are means ± standard error from three replicates. Means followed by same small letters (a, b, c, d) with in the column are not significant at *P* ≤ 0*.*05. CK: control, Q: 0.25 g/L quinclorac, S: 10 mg/L SA followed by 0.25 g/L quinclorac

The aboveground parts of the two rice cultivars were used for analysis. Quinclorac application triggered enhancements of antioxidant enzyme activities except catalase (CAT) in comparison with the control (Fig. 3A). SA pre-treatment (S) further enhanced the activity of all enzymes compared to quinclorac treatment alone (Q). Superoxide dismutase (SOD), ascorbate peroxidase (APX) and peroxidase (POD) activities showed the same increasing tendency in the leaves of rice. The content of MDA was highly accumulated under quinclorac stress while SA significantly reduced it (Fig. 3B). Regarding reactive oxygen intermediates including hydroxyl radicals (OH^−^) and hydrogen peroxide (H_2_O_2_), the similar response occurred in the two cultivars as well. The varied trend of soluble protein was consistent with CAT (Fig. 3A, B). After the application of quinclorac, reduced glutathione (GSH), oxidized glutathione (GSSG) and glutathione reductase (GR) were found to be lower compare to control (Fig. 3C). Meanwhile, SA helped these enzymes to return the normal level or even higher. In a word, there were significant differences among the three different groups (CK, Q, S) according to the results of physiological parameters measured in leaves.

### Spectral features

**Fig. 4:**
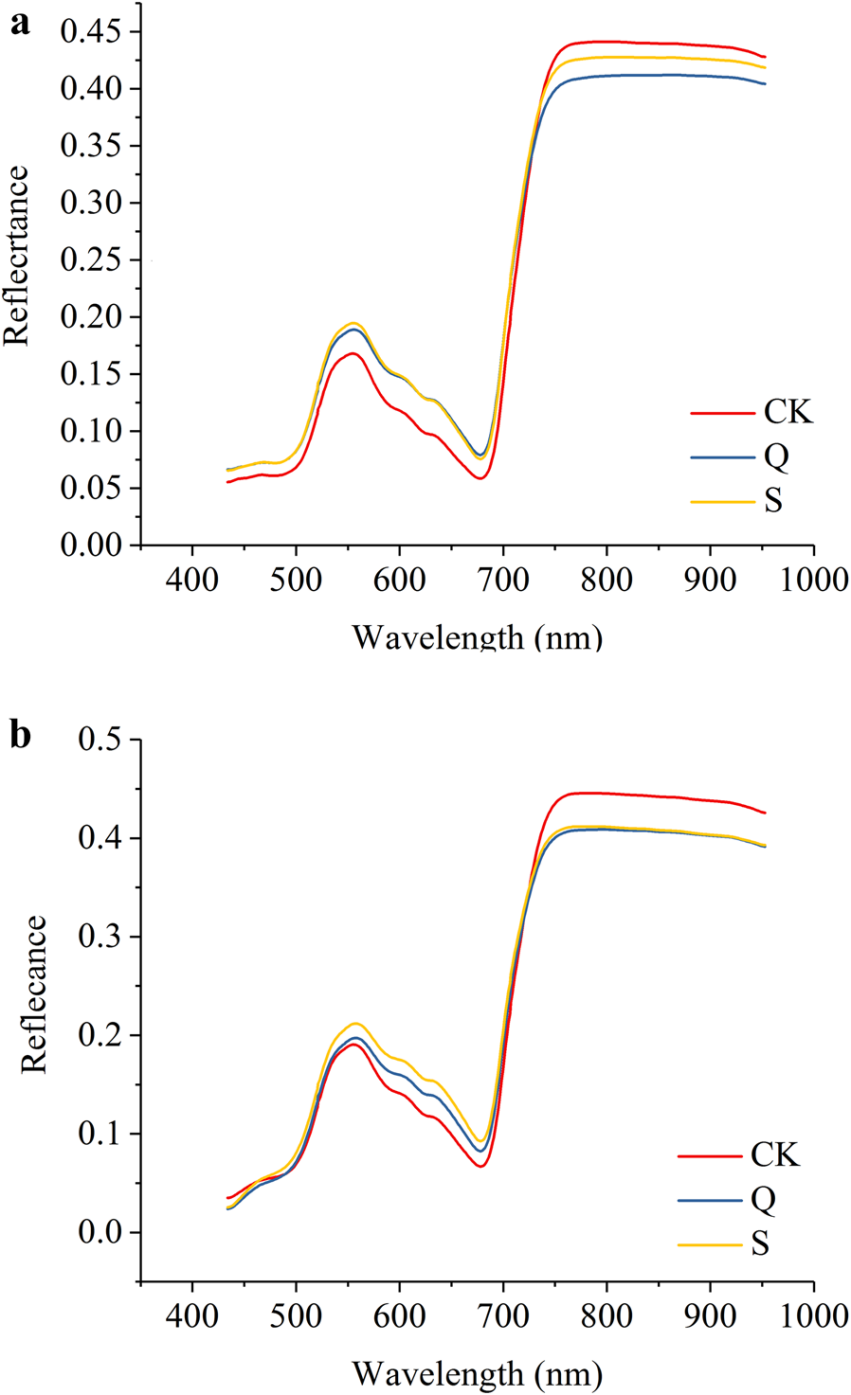
Average spectra of the three groups of XS 134 (**a**) and ZJ 88 (**b**). CK: treated with nutrient solution; Q: treated with 0.25 g/L quinclorac, S: pre-treated with 10 mg/L SA under 0.25 g/L quinclorac stress

The average spectra for the aboveground samples are presented in Fig. 4. Typical spectra of plant leaves could be observed. For the two rice cultivars, differences among each group (CK, Q, S) could be observed. The spectra of CK group showed larger differences from the other two groups, while the spectra of the Q and S groups were much closer. The reflectance between 434 and 700 nm of the CK group was lower than that of the Q and S groups. The reflectance spectra between 434 and 700 nm are related to the leaf pigments, according to our previous study [7]. Leaf pigment content increased with the decrease of quinclorac content. The reflectance spectra matched with the spectra in ref [56]. Moreover, the reflectance between 750 and 953 nm of the CK group was higher than that of the Q and S groups. Studies have showed that reflectance spectra were less sensitive in the near-infrared region to stresses [57]. In the region of 700–1300 nm, leaf reflectance is governed by their cellular structure [58]. Similar phenomenon could be found for rice leaves under the stress of arsenic stress [59], diseases [60], rice leaf folder [61] and under heat treatment [62], etc. The samples were divided into two data sets at a ratio of 2:1 for calibration and prediction according to their groups. The CK group, the Q group and the S group were assigned the category value of 1, 2 and 3 for modelling, respectively.

### PCA score images analysis

**Fig. 5:**
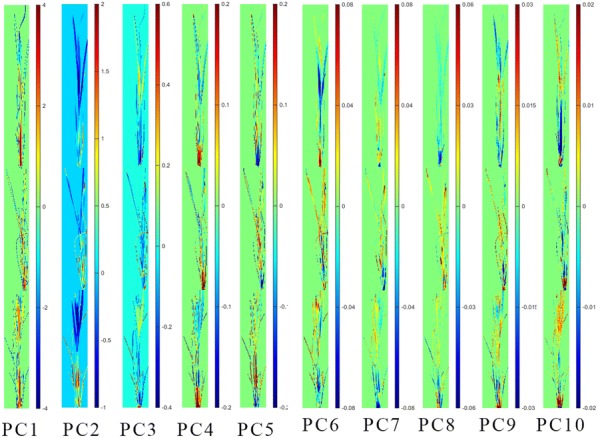
PCA score images from PC1 to PC10 of the three groups of XS 134. CK: treated with nutrient solution, Q: treated with 0.25 g/L quinclorac, S: pre-treated with 10 mg/L SA followed by 0.25 g/L quinclorac

**Fig. 6:**
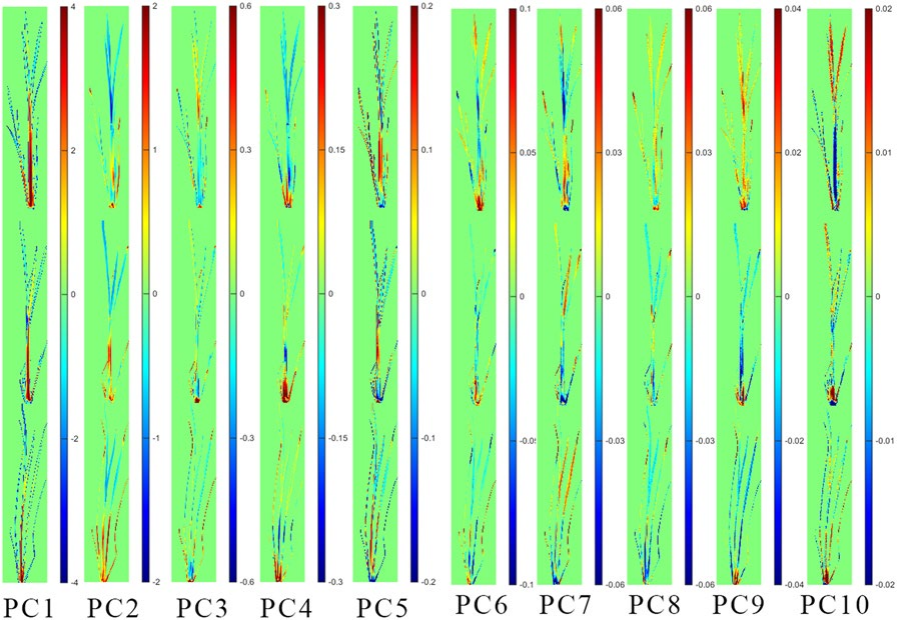
PCA score images from PC1 to PC10 of the three groups of ZJ 88. CK: treated with nutrient solution, Q: treated with 0.25 g/L quinclorac, S: pre-treated with 10 mg/L SA followed by 0.25 g/L quinclorac

For each rice cultivar, there were three groups of samples under different treatments. PCA was firstly applied to form score images at different PC to qualitatively analyze the differences among samples under different treatments. One image was randomly selected from each group, and PCA was conducted on the pixel-wise spectra obtained by the hyperspectral imaging system to evaluate the difference among the groups. The first ten principal components (PCs) of XS 134 and ZJ 88 both explained over 99.99% of total variance (Figs. 5 and 6). The PCA score images from PC1 to PC10 were shown in Figs. 5 and 6. Score values were presented in pseudo color.

Figure 5 shows the PCA score image of XS 134. Differences on the color distributions were observed in the PCA scores images from PC1 to PC10. In general, images of PC1, PC5, PC6, PC7 and PC9 showed that the groups of Q and S were close and they were different from the CK group. Images of PC2, PC3 and PC4 showed that groups of CK and S were close, and they were different from the Q group. Images of PC8 and PC10 showed that there were differences among the CK, Q and S groups.

Figure 6 shows the PCA score image of ZJ 88. Differences within PC1 image were small. Images of PC3 showed differences among the CK, Q and S groups. Images of PC2, PC4, PC6, PC7, PC8, PC9 and PC10 showed that groups of Q and S were close and they were different from the CK group.

The PCA score images indicated that there were differences among the CK, Q and S groups of the two rice cultivars. Specially, differences between the CK group and Q group were larger, while differences between the CK group and S group, Q group and S group were smaller.

### SVC models using full spectra

To evaluate the effect of herbicides on rice plants, SVC models were furtherly built by using the full spectra. To find the optimal SVC model, the model parameters (penalty coefficient *C* and RBF kernel parameter *g*) were optimized by a grid-search procedure. Here we separated the datasets into three different categories (training, validation and test sets) at the ratio of 4:1:1. To build SVC models, the penalty coefficient (*C*) and kernel parameter (*g*) were optimized by a grid-search procedure. It was quite difficult to determine the grid for SVC models, and the range of *C* and *g* was 10^ N^ (N = − 8, − 7, − 6,…, 6, 7, 8, the step of N is 1) based on trails and experiences. The optimal SVC model was determined by the best classification accuracy of training and validation. The discriminant results of SVC models using the full spectra of the aboveground segments of rice cultivars XS 134 and ZJ 88 are shown in Table 2. Performances of SVC models were evaluated by classification accuracy and kappa coefficient.

**Table 2:** Confusion matrix of SVC models using full spectra

Cultivar	Parameter^a^		Training set					Validation set					Testing set				
			CK	Q	S	Accuracy^b^	Kappa^c^	CK	Q	S	Total^b^	Kappa^c^	CK	Q	S	Accuracy^b^	Kappa^c^
XS 134	(10^7^,10^–2^)	CK	25	0	0			6	0	0			5	0	1		
		Q	0	23	3			0	6	0			0	7	0		
		S	1	1	26			0	2	5			1	0	6		
						93.67%	90.47%				89.47%	80.65%				90%	84.96%
ZJ 88	(10^7^, 10^–2^)	CK	28	0	0			7	0	0			6	0	0		
		Q	0	28	0			0	6	1			0	6	0		
		S	0	0	26			0	0	6			0	0	7		
						100%	100%				95%	92.51%				100%	100%

^a^Parameter means the model parameter of SVC, which is the combination of penalty coefficient C and RBF kernel parameter g, i.e. (C, g)

^b^Total means the total classification accuracy

^c^Kappa is used to evaluate the inter-rater reliability of the classification results

CK: treated with nutrient solution, Q: treated with 0.25 g/L quinclorac, S: pre-treated with 10 mg/L SA followed by 0.25 g/L quinclorac

For XS 134, the good classification results were obtained with the accuracy of three datasets approaching or over 90%. No samples from CK and Q groups were misclassified with each other. Samples from the Q group and the S groups were more likely to be misclassified as each other, and samples from the CK group and the S group were more likely to be misclassified as each other. In the training set, three samples from the Q group were misclassified as samples in the S group. Two samples from the S group were misclassified as samples in the CK group and the Q group. Two samples from the S group in the validation set were misclassified as the samples in the Q group. In the test set, one sample from the CK group and the S group was misclassified as each other.

For ZJ 88, the samples from different groups could be classified precisely. Compared with XS 134, better classification results were obtained, with classification accuracy of the training and test set equaling to 100%. Only one sample from the Q group in the validation set was misclassified as the sample from the S group.

The SVC model of ZJ 88 performed better than that of XS 134. The reason can be attributed to that XS 134 was relatively resistant to herbicide quinclorac, and the effect of SA was not as significant as in ZJ 88. ZJ 88 was relatively susceptible to herbicide quinclorac, and SA worked better after being added to the rice plants under quinclorac stress.

## SVC models using extracted features

PCA was also used for feature extraction. As mentioned above, the first ten PCs explained over 99.99% of total variances, and the scores of the first ten PCs were extracted as features. To evaluate the performance of extracted features, the first ten PCs were used as inputs of SVC models. The results were shown in Table 3.

**Table 3:** Confusion matrix of SVC models using extracted features

Cultivar	Parameter^a^		Training set					Validation set					Testing set				
			CK	Q	S	Accuracy	Kappa	CK	Q	S	Total	Kappa	CK	Q	S	Accuracy	Kappa
XS 134	(10^5^, 10^–1^)	CK	25	0	0			6	0	0			5	0	1		
		Q	0	21	5			0	6	0			0	6	1		
		S	0	2	26			0	2	5			1	0	6		
						91.14%	86.68%				89.47%	80.65%				85%	76.15%
ZJ 88	(10^6^, 10^–1^)	CK	28	0	0			7	0	0			6	0	0		
		Q	0	28	0			0	6	1			0	6	0		
		S	0	0	26			0	0	6			0	2	5		
						100%	100%				95%	92.5%				89.47%	80.5%

^a^Parameter means the model parameter of SVC, which is the combination of penalty coefficient C and RBF kernel parameter g, i.e. (C, g)

^b^Total means the total classification accuracy

^c^Kappa is used to evaluate the inter-rater reliability of the classification results

CK: treated with nutrient solution, Q: treated with 0.25 g/L quinclorac, S: pre-treated with 10 mg/L SA followed by 0.25 g/L quinclorac

For XS 134, the classification accuracy of the training set and the validation set of the aboveground segments fluctuated around 90%, while the accuracy of testing set is a little bit lower. Most of the inaccuracies existed between the Q and S groups. Similar phenomenon was observed in case of ZJ 88. The division accuracies of all datasets were elevated in ZJ 88. Only one sample from the Q group was mismatched as the sample from the S group in the validation set.

**Fig. 7:**
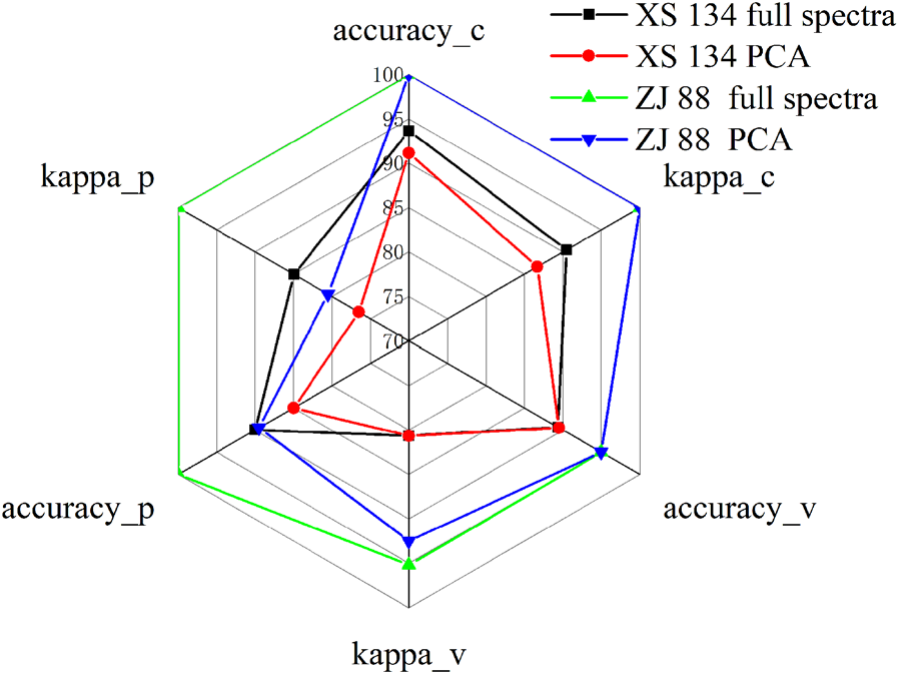
The accuracies and kappa of the models using full range of spectral response and PCA. *c* calibration, *v* validation, *p* prediction

Compared with SVC models using full spectra, SVC models using extracted features showed slightly worse results. A radar graph presenting the classification results is shown in Fig. 7. As shown in Fig. 7, Tables 2 and 3, the SVC model using full spectra of ZJ 88 obtained the better performance, the SVC model using extracted features of XS 134 obtained the worse results. The overall results indicated that the cultivar ZJ 88 exhibited greater differences and was easier to be classified than XS 134. The results also corroborated the cultivar characteristics of ZJ 88 (relatively susceptible to herbicide quinclorac) and XS 134 (relatively resistant). Moreover, the number of input variables of SVC models reduced from 410 to 10 after feature extraction, resulting in a reduction of 97.56%. The results showed that the extraction of spectral features which contributed mostly to the evaluation of quinclorac stress of rice plants was of great value.

### Image visualization

**Fig. 8:**
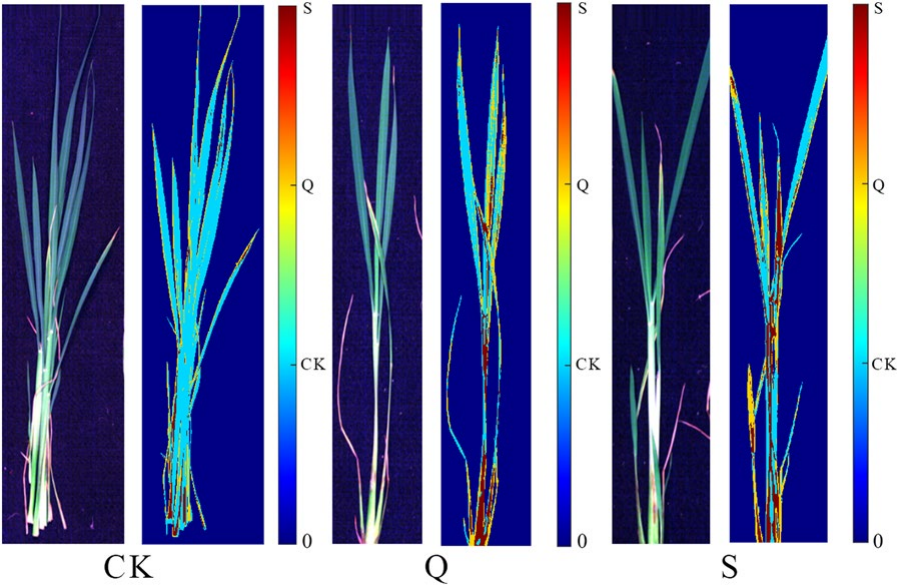
Original RGB images and the distribution maps of the leaves of XS 134. CK: treated with nutrient solution, Q: treated with 0.25 g/L quinclorac, S: pre-treated with 10 mg/L SA followed by 0.25 g/L quinclorac

**Fig. 9:**
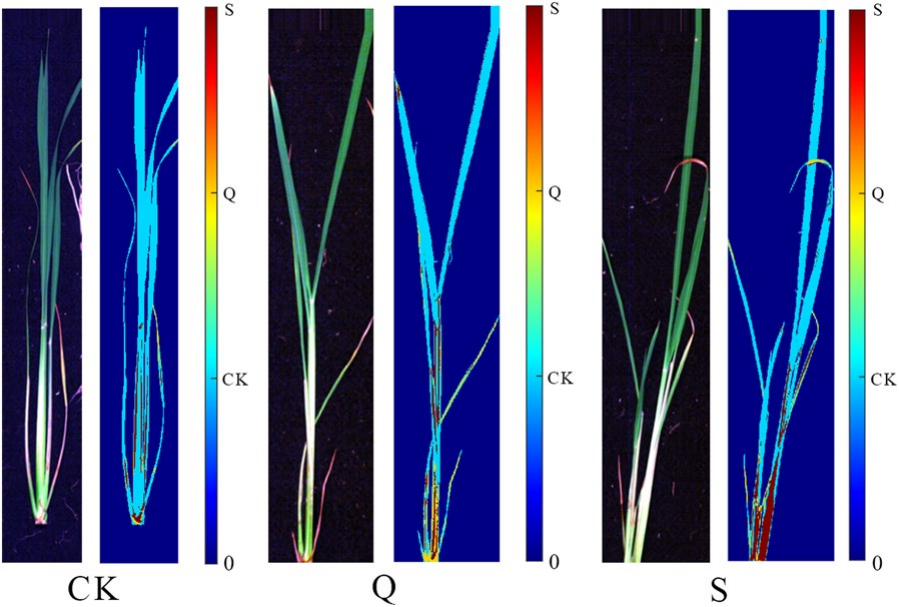
Original RGB images and the distribution maps of the leaves of ZJ 88. CK: treated with nutrient solution, Q: treated with 0.25 g/L quinclorac, S: pre-treated with 10 mg/L SA followed by 0.25 g/L quinclorac

As the SVC models using features extracted by PCA exhibited good classification performance, hence they were used to predict the features of each pixel to form the prediction maps. The prediction maps of one randomly selected aboveground segments of XS 134 and ZJ 88 under different treatments were shown in Figs. 8 and 9. In the prediction maps, pseudo colors were used to represent the sample status.

As shown in Figs. 8 and 9, differences among the three groups were observed. For better understanding the prediction maps, predicted values of pixel-wise spectra of all samples are summarized in Table 4.

**Table 4:** Summary of pixel-wise predicted mean values of all samples for image visualization of XS 134 and ZJ 88

Cultivar	Treatment	CK	Q	S
XS 134	CK	72.6% ± 5.7%a	17.3% ± 3.9%c	10.1% ± 3.3%b
	Q	50.4% ± 4.8%c	29.9% ± 5.9%a	19.7% ± 4.5%a
	S	53.9% ± 5.3%b	25.3% ± 5.0%b	20.8% ± 5.3%a
ZJ88	CK	86.7% ± 3.5%a	3.4% ± 1.3%c	9.9% ± 2.8%b
	Q	74.2% ± 8.1%b	10.0% ± 4.7%a	15.8% ± 4.6%a
	S	76.5% ± 5.6%b	7.7% ± 2.6%b	15.8% ± 3.9%a

The percentage denotes the ratio of pixels predicted as each category. Means ± SD followed by same letters (a, b, c) in the vertical column are not significant at P ≤ 0.05

CK: treated with nutrient solution, Q: treated with 0.25 g/L quinclorac, S: pre-treated with 10 mg/L SA followed by 0.25 g/L quinclorac

As for XS 134 in Table 4, pixels were predicted as the CK, Q and S groups in each sample. For samples from the CK, Q and S groups, the percentage of pixels predicted as CK showed significant differences, the percentage of pixels predicted as Q also showed significant differences. The percentage of pixels predicted as S in the samples from Q and S were close. As for ZJ 88 in Table 4, pixels were predicted as the CK, Q and S groups in each sample. For samples from the CK, Q and S groups, the percentage of pixels predicted as Q showed significant differences. The percentage of pixels of samples from Q and S predicted as CK as well as S were close.

Different performances of XS 134 and ZJ 88 could be observed in Table 4, the differences were caused by the sample themselves and the model performances. It should be noted that the number of samples used in this study was small, and in future studies, more samples with wider sample ranges should be studied. An important phenomenon was also found that the pixels predicted as CK was dominant in the three prediction images of the two rice cultivars. The reason can be attributed to that different parts have different responses to the stresses. The prediction maps indicated that hyperspectral imaging was effective in monitoring rice plants under the stress of quinclorac toxicity.

## Discussion

In the analysis of physiological parameters, SOD is an important enzyme and is considered as a first line of defense in plants under stress conditions. APX is a vital enzyme related to the regulation of reactive oxygen intermediates (ROIs) for signaling, and CAT plays a crucial role in reactive oxygen scavenging systems under stress [63]. Then the high accumulation of hydrogen peroxide resulted in the consumption of CAT content (Fig. 2, c and g). POD up-regulation after herbicide application has been reported in maize, tobacco and many other crop species [64]. MDA is used as an index of lipid peroxidation. When plants are expose to the herbicides, the accumulation of toxic ROS such as OH^−^, H_2_O_2_, which causes damage to lipids, proteins and nucleic acids and leads to rapid plant death [65]. In the present experiment, the MDA, OH^−^, H_2_O_2_ and soluble protein contents were highly induced after herbicide treatment (Fig. 2e–g). Previously, H_2_O_2_ overproduction in cleavers (*Galiumaparine* L.) caused tissue damage under auxin herbicides toxicity [20]. SA-induced DELLAs accumulation has been found to increase the gene expression of detoxifying enzymes, thereby reducing the ROS levels [66]. Consistent results of ROS changes were observed in our study (Fig. 3F and G). These findings are suggesting that SA allow the restoration of redox homeostasis under quinclorac stress condition. GSH exists in living cells and is involved in various physiological and biochemical reactions. GSSG is reduced to GSH by glutathione reductase GR. GSH is oxidized to GSSG by glutathione peroxidase and dehydroascorbate reductase through the process of scavenging H_2_O_2_ [63]. GSH plays a crucial role in herbicide detoxification [67]. In this study, GSH was decreased to protect the plant from quinclorac toxicity (Fig. 3I). SA helped the rice to synthesize more GSH to strengthen its defense system. Elevated concentrations of GSSG are the evidence of oxidative stress in plants [67]. SA induced GSSG to increase along with GSH conversion compared to the leaves under quinclorac stress. This may be a result of GSSG converting quickly to GSH to maintain the detoxifying process. The GR enzyme varied according to the balance between GSH and GSSG. The changes in enzymatic and non-enzymatic antioxidants and modulation of ROS demonstrated that there were noticeable differences among CK, Q and S groups. It provides the possibility to establish the model to quickly separate the rice seedlings under quinclorac stress or recovery stage with SA pre-treatment in an indirect way.

Except for physiological parameters, optical properties can reveal the internal and external features of plants. The unique characteristic of hyperspectral imaging to obtain spectrum of each pixel makes it quite efficient and convenient to analyze not only the optical features and morphological features, etc. In this study, hyperspectral images were acquired for analysis based on optical features of rice. Reflectance spectra indicated that there were differences of rice seedlings among the three different treatments. PCA score images showed the feasibility of using hyperspectral imaging to explore the differences among different herbicide stresses, and the results confirmed that the rice seedling changed with the herbicide stresses. Furthermore, the established SVC models showed the good performances of the detection of rice seedlings under the stress of quinclorac toxicity and its alleviation by salicylic acid. These results proved the feasibility that hyperspectral imaging could be used as an effective, rapid and non-destructive tool to monitor rice seedlings under stress of herbicides and its alleviation by salicylic acid. Additionally, hyperspectral imaging provided a promising alternative with significant potential in monitoring plant growth.

Symptoms of herbicide injury at early stages are difficult to recognize. Researchers have used the visible and near-infrared spectroscopy to study plants under the stresses of herbicide. Tian et al. [68] used near-infrared spectroscopy to study oilseed rape under the stress of propyl 4-(2-(4,6-dimethoxypyrimidin-2-yloxy) benzylamino)benzoate (ZJ0273), and determined the total- and branched-chain amino acids in oilseed rape under the stress of ZJ0273 herbicide. Liu et al. [32, 69] applied near-infrared spectroscopy to determine acetolactate synthase activity and total amino acids in oilseed rape under the stress of herbicide. Bao et al. [70] utilized near-infrared spectroscopy to determine total amino acids content in barley leaves under the stress of herbicides. As for hyperspectral imaging, not much work has been done. Kong et al. [71] used hyperspectral imaging to determine and visualize malondialdehyde (MDA) distribution in oilseed rape leaves under the stress of herbicide. Our work showed the feasibly of using hyperspectral imaging to study phenotypes of rice seedlings under the stress of herbicides. In future, different kinds of herbicides should be studied, and the differences of phenotypic variation between the herbicide stresses and other stresses should also be clarified. The use of hyperspectral imaging in rice plant phenotyping should be further extended and explored.

SVC has been widely used in spectral data analysis. Hyperspectral images could be acquired at the spectral range of 400–2500 nm. In this study, the analyzed spectral range is 434–953 nm. This spectral range relates to the pigments and leaf or canopy structure [44–48]. The spectra at the range of 1000–2500 nm are near-infrared region which relates to the chemical compositions. The sub-ranges of 400–2500 nm have been widely studied in plant science [72]. SVC has also been used in various studies based on different spectral ranges. Indeed, SVC is a machine learning method with unique characteristics. By using spectral information with SVC for rice herbicide stress monitoring, the spectral information relating to changes of plants under the stress is the basis for stress monitoring. SVC extracts the inner features from the obtained spectral data and uses these features to make decisions. For different spectral ranges, the spectral data contain different information reflecting sample status, and the performances of SVC models essentially depend on the information. Moreover, under the stress of herbicides, the chemical compositions will change, and the spectral data in the range of 1000–2500 nm reflecting the chemical compositions is feasible to be used to monitor rice herbicide stress. SVC is possible to obtain good performances at different spectral ranges in which the spectral information relating the stress is contained.

## Conclusion

Hyperspectral imaging covering a spectral range of 380–1030 nm was used to evaluate herbicide quinclorac phytotoxicity on rice plants. The aboveground segments of two rice cultivars were studied for physiological changes to quinclorac toxicity detection and for imaging calibration and prediction. PCA was used to form score images and extract features. Full-spectra-based SVC models and extracted-feature-based SVC models all achieved good performance. The SVC models using optimal wavelengths were applied to hyperspectral images to obtain prediction maps. The SVC models and prediction maps showed feasibility of using hyperspectral imaging to evaluate the herbicide stress on rice plants. The classification performances of ZJ 88 were better than those of XS 134, thus indicating differences between the two cultivars. Hyperspectral imaging is a promising technique for monitoring herbicide stress at least for quinclorac on rice plants. For the subsequent study considerations based on the current results, it is recommended that more measurement time intervals should be used in the first 24 h of herbicide application.

## Materials and methods

### Plant materials and preparation

Two Japonica rice (*Oryza sativa* L.) cultivars: i.e., one relatively susceptible (cv. ZJ 88) and one relatively resistant (cv. XS 134) to herbicide quinclorac, were used in this experiment [7]. The healthy seeds were first surface sterilized in 75% ethanol for 5 min and 0.1% NaClO for another 15 min and then soaked in distilled water for an additional 20 min. A total of fifty seeds of each group were sown in plastic germination boxes with wet double filter papers. Germination was manipulated at 30 °C for 48 h. The germinated seedlings were selected and kept in darkness for two days and then cultured in a growth chamber with available areas of 1.5 m^2^, constant day/night temperatures of 25/20 °C, programmed for a 14-h photoperiod, with a mixed incandescent and fluorescent irradiance of 300 μmol m^−2^ s^−1^, and a relative humidity of 70–80%.

The samples in the pot (16 cm diameter × 15 cm height) were divided into three groups including the control group (the CK group), the quinclorac treatment group (the Q group), and the group pre-treated with SA and followed by quinclorac stress (the S group). A total of 0.25 g/L of quinclorac (with 10% active ingredient, in the form of wet powder) was treated in a solution at the four-leaf stage. SA at 10 mg/L was added to the solution 48 h before the quinclorac treatment. Each treatment was replicated three times, and each replication with approximately 40 plants. The concentrations of different treatments were based on previous experimental data. The results of herbicide experiments are presented in Additional file 1.

The nutrient solution was renewed every five days with a Hoagland solution. The plants were grown under hydroponic condition. Ten days after treatment, the leaf samples were first prepared for hyperspectral image acquisition. Later, plant samples for biochemical analyses were collected. A total of 239 samples were prepared (121 samples for ZJ88 and 118 samples for ZJ 88) for the treatments. Two-thirds of the plants were selected as the calibration set using the Kennard-Stone algorithm [73], and the remaining samples were used as the prediction set.

### Measurement of physiological parameters

#### Measurement of lipid peroxidation and antioxidant enzyme activities

Malondialdehyde (MDA) was measured as an indicator of lipid peroxidation. Lipid peroxidation was determined by using according to Zhou and Leul with some modifications [74]. Superoxide dismutase (SOD) activity was assayed as described by Zhang et al. [75]. Peroxidase (POD) activity was determined as described by Zhou and Leul [74]. Ascorbate peroxidase (APX) activity was assayed following Nakano and Asada [76] with a reaction of H_2_O_2_.

#### Measurement of reactive oxygen species and total soluble protein

Hydrogen peroxide (H_2_O_2_) was estimated using the method by Velikova et al. [77] and Halliwell and Gutteridge [78] with some modifications. The soluble protein concentration was determined according to the method of Bradford [79].

#### Measurement of non-enzymatic antioxidants

Glutathione GSH and GSSG were analyzed using the method of Law et al. [80]. Glutathione reductase (GR) activity was assayed according to Jiang and Zhang [81].

### Statistical analysis

All the treatments were arranged in a completely randomized block design. Biochemical data i.e. the changes of enzymes activity were presented as mean values of three replicates ± standard error. The data were analyzed using a statistical package, SPSS (Version 19.0). One-way analysis of variance was employed followed by Duncan’s multiple range test to determine the significant differences among means of the treatments at 5% level of significance.

### Hyperspectral imaging system and image acquisition

A ground-based visible and near-infrared hyperspectral imaging system (spectral range: 380–1030 nm; spectral resolution: 2.8 nm) was used to acquire hyperspectral images. An imaging spectrograph (ImSpector V10E; Spectral Imaging Ltd., Oulu, Finland), a high performance CCD camera (Hamamatsu, Hamamatsu City, Japan), a camera lens (OLES23; Specim, Spectral Imaging Ltd., Oulu, Finland), two 150 W tungsten halogen lamps for line illumination (Fiber-Lite DC950 Illuminator; Dolan Jenner Industries Inc., Boxborough, MA, USA), and a sample motion plate to move the samples (Isuzu Optics Corp, Taiwan, China) were used to assemble the system. The illumination lamps were placed symmetrical on both sides of the camera with the angle of 45°. The hyperspectral imaging system acquires images at a spectral resolution of 2.8 nm. This hyperspectral imaging conducted line scan, and the line light were focused on the place directly under the camera lens. Sample moving speed, camera exposure time, and the lens height to the sample were adjusted to 2.85 mm/s, 0.09 s, and 35 cm respectively for image acquisition. The acquire image size was 672 pixels (width) × *L* (pixels) × 512 (wavebands), where *L* was number of pixels in the length of the image.

The original raw images were corrected to reflectance hyperspectral images for further processing. The calibrated image *Ic* was calculated using the following equation [72]:

1 $$I_{c} = \frac{{I_{raw} - B}}{W - B}$$

where *I*_*raw*_ was the raw hyperspectral image, *B* was the dark reference image acquired with reflectance close to 0, *W* was the white reference image acquired with reflectance close to 100%, and *Ic* was the calibrated hyperspectral image.

### Spectra extraction

After image correction, spectra data were extracted from hyperspectral images. The aboveground segments were used for analysis. The entire aboveground segment was defined as the region of interest (ROI). Pixel-wise spectra within the ROI were extracted. Because there was obvious random noise in the origin spectra, only the spectra at the range of 434–953 nm were studied. Wavelet transform (wavelet function Daubechies9 with decomposition level 3) followed with the moving average smoothing with 7 smoothing points was applied to preprocess the pixel-wise spectra. Average spectrum calculated from the preprocessed pixel-wise spectra within each ROI was used as sample spectrum.

### Data analysis methods

#### SVC

The SVC method is based on a computer algorithm that learns by example to assign labels to objects, and has been widely used for supervised pattern recognition in various biological applications [82]. The SVC has been applied in chemo-metrics [83], NIR classification tasks such as material identification [84, 85] and food discrimination [86–88]. The general concept of SVC is to transform the origin data into a higher dimensional space where the samples are more likely to be linearly separable. The optimal hyperplane is constructed to maximize the shortest distances between the samples of each class in the higher dimensional space. The distance defines the margin associated to the separating hyperplane [89]. Selection of kernel function is a pivotal factor which determines performance of SVC [90]. Among existing kernel functions, the radial basis function (RBF) as a kernel function has been proven to be efficient and good performance is generally obtained [91]. So RBF was used for the SVC models in this study. The brief introduction of SVC is presented as follow.

First, we define the symbols and parameters used in the mathematical theory of the SVC algorithm as Table 5.

**Table 5:** The symbols and parameters used in the mathematical theory of the SVC algorithm

Parameter	Description
*w*	The normal direction of the hyperplane
*b*	The bias of the hyperplane
*ξ*_*i*_	The positive slack variable
*C*	The user-defined parameter to assign penalty to errors
*e*	The vector of all ones
*α*_*i*_	The Lagrangian multipliers
K(*x*_*i*_,*x*_*j*_)	The kernel function
*γ*	The tuning parameter (called ‘*gamma*’) of the RBF kernel, must be greater than 0

Given training dataset *S* = {*X*,*Y*} in two classes, where *X* = {*x*_*1*_, *x*_*2*_,…,*x*_*n*_}, *Y* = {*y*_*1*_, *y*_*2*_,…,*y*_*n*_}, vectors $x_{i} \in R^{p} ,i = 1,2, \ldots ,n$, and vector $y \in \left\{ {1, - 1} \right\}^{n}$. SVC method uses a hyperplane maximizing the margin border of different sample points. The hyperplane is constructed as:

2 $$w^{T} X + b = 0$$

For non-separable data, SVC solves the following primal problem:

3 $$\mathop {\min }\limits_{w,b,\zeta } \left( {\frac{1}{2}w^{T} w + C\sum\limits_{i = 1}^{n} {\zeta_{i} } } \right)$$

The constraints of Eq. (3) are:

4 $$\begin{gathered} yi(w^{T} \phi (x_{i} ) + b) \ge 1 - \zeta_{i} , \hfill \\ \zeta_{i} \ge 0, \quad i = 1,2, \ldots ,n \hfill \\ \end{gathered}$$

Equation (4) can be transformed to its dual problem under the conditions of Karush–Kuhn–Tucker:

5 $$\begin{gathered} \min \left( {\frac{1}{2}\alpha^{T} Q\alpha - e^{T} \alpha } \right) \hfill \\ Q_{ij} = y_{i} y_{j} K(x_{i} ,x_{j} ) = y_{i} y_{j} \phi (x_{i} )^{T} \phi (x_{j} ) \hfill \\ \end{gathered}$$

The constraints of Eq. (5) are:

6 $$\begin{gathered} y^{T} \alpha = 0, \hfill \\ 0 \le \alpha_{i} \le C,\quad i = 1,2, \ldots ,n \hfill \\ \end{gathered}$$

In this study, the popular radial basis function (RBF) was used as the kernel function which can be expressed as:

7 $$K(x_{i} ,x_{j} ) = \exp \left( { - \gamma \left\| {\left. {x_{i} - x_{j}} \right\|^{2} } \right.} \right)$$

Then the decision function can be expressed as:

8 $$f(x) = sign\left( {\sum\limits_{i = 1}^{n} {y_{i} a_{i} \exp \left( { - \gamma \left\| {\left. {x_{i} - x} \right\|} \right.} \right) + b} } \right)$$

The C and gamma are the two parameters which should be optimized. Only the binary SVC classifiers for two classes’ situations are introduced in this section, it is easy to extend to multi-class issue.

#### PCA

PCA is an unsupervised classification method which reduces the dimensionality of the data while keeping most of the variation in the data set, and PCA is generally used for qualitative analysis [92]. PCA linearly transforms the original data into new orthogonal variables PCs [93]. Then each sample can be plotted with a few components, making it possible to represent the similarities and differences visually and demonstrate whether samples can be grouped [94]. Generally, the first few PCs carry most of the information that is used for qualitative analysis to visualize the sample clusters in the score spaces. In hyperspectral image analysis, PCA is also applied to pixels within the hyperspectral images to form the score visualization image. Based on the score images of different, variations of spectral information within one sample or among different samples can be observed. A color gradient representing the scores values can help to visualize the differences. In this study, PCA was firstly applied to pixels within the hyperspectral images to explore the differences among rice leaves under different quinclorac stresses prior to building classification models.

### Feature extraction

Feature extraction is a widely used approach to extract the useful features from the original data. Feature extraction conducts the data transformation to transform the original features into new features. New features contain the most of the useful information can be used instead of the original data. Use of the extracted features can reduce the amount and redundancy of the data, simplify the model, and improve the model performance and model stability.

PCA, as mentioned above, is a widely used feature extraction method [95]. Generally, the first few PCs explain the most of the variance and contain the most of useful information. Scores of these first few PCs can be used as new features to represent the features of the original data. Then the scores can be used to build multivariate models.

In this study, PCA was firstly applied to pixel-wise spectra within hyperspectral images to form score images for qualitative analysis of differences among samples under different treatments. After building SVC models using full average spectra, PCA was used to extract features for data dimension reduction, and SVC models were built using the extracted features. The performances of SVC models using full spectra and extracted features were compared.

### Image visualization

In hyperspectral images, each pixel contains a spectrum, which makes it possible to use the established model to predict the pixels within the hyperspectral images to form a prediction map (called image visualization) [96, 97]. The brief procedure for image visualization is as follows:

1) Extracting the spectral data from hyperspectral images;

2) Establishing calibration models;

3) Developing a prediction map by applying the established models to each pixel.

Image visualization provides direct visual presentation of the sample features and their distribution. It would help to present the variant distribution of different features within samples. In general, the prediction map is presented in colors, and the colors represent the corresponding feature values.

### Software and model evaluation

The image segmentation to isolate the rice leaves from the background were conducted with ENVI 4.6 (ITT, Visual Information Solutions, Boulder, Co., USA). The spectral data extraction after image segmentation, model establishment and image visualization were conducted on Matlab R2014b (The Math Works, Natick, MA, USA). SVC was conducted using libSVC toolbox (version 3.1) [98]. Software is available at https://www.csie.ntu.edu.tw/~cjlin/libSVC. The classification accuracy was used to evaluate the calibration and prediction performances of the classification models.

## Supplementary information

**Additional file 1: Table S1.** Effects of different treatments of quinclorac herbicide and salicylic acid (SA) on biomass (g) and total chlorophyll [mg g-1(FM)] of two rice cultivars.

## Data Availability

Not applicable.
