# Supplementary material for: Evaluation of quinclorac toxicity and alleviation by salicylic acid in rice seedlings using ground-based visible/near-infrared hyperspectral imaging
Source: Plant Methods. 2020 Mar 5;16:30. doi: 10.1186/s13007-020-00576-7 (PMC7059665; doi:10.1186/s13007-020-00576-7)
Supplement: Supplementary file 1 — Additional file 1: Table S1. Effects of different treatments of quinclorac herbicide and salicylic acid (SA) on biomass (g) and total chlorophyll [mg g-1(FM)] of two rice cultivars. [file 13007_2020_576_MOESM1_ESM.docx]

**Table S1** Effects of different treatments of quinclorac herbicide and salicylic acid (SA) on biomass (g) and total chlorophyll [mg g^-1^(FM)] of two rice cultivars.

| Cultivar | SA conc. (mg/L) | Quinclorac conc. (g/L) | Leaf FW  (5 plants) | Root FW  (5 plants) | Leaf DW  (5 plants) | Root DW  (5 plants) | Total Chl |
| --- | --- | --- | --- | --- | --- | --- | --- |
| XS 134 | 0 | 0 | 3.56±0.25a | 1.50±0.10a | 0.48±0.03ab | 0.13±0.02a | 1.94±0.12a |
|  |  | 0.1 | 3.45±0.14a | 1.42±0.16ab | 0.43±0.02c | 0.11±0.02bc | 1.83±0.14ab |
|  |  | 0.5 | 2.78±0.28b | 0.89±0.15c | 0.23±0.02e | 0.07±0.01d | 1.07±0.24c |
|  | 10 | 0 | 3.57±0.17a | 1.49±0.08a | 0.49±0.03a | 0.14±0.01a | 1.93±0.12a |
|  |  | 0.1 | 3.51±0.09a | 1.45±0.07ab | 0.45±0.02bc | 0.13±0.02ab | 1.87±0.13a |
|  |  | 0.5 | 3.23±0.12a | 1.23±0.16b | 0.35±0.01d | 0.10±0.01c | 1.58±0.13b |
| ZJ 88 | 0 | 0 | 3.55±0.13a | 1.49±0.13a | 0.46±0.02ab | 0.12±0.02a | 1.89±0.13a |
|  |  | 0.1 | 3.35±0.14a | 1.42±0.17a | 0.41±0.03c | 0.10±0.01b | 1.72±0.13a |
|  |  | 0.5 | 2.20±0.26c | 0.67±0.12c | 0.13±0.02e | 0.04±0.01d | 0.52±0.11c |
|  | 10 | 0 | 3.56±0.22a | 1.53±0.12a | 0.48±0.01a | 0.14±0.02a | 1.91±0.12a |
|  |  | 0.1 | 3.38±0.14a | 1.46±0.23a | 0.43±0.02bc | 0.12±0.02a | 1.93±0.13a |
|  |  | 0.5 | 2.55±0.14bc | 0.95±0.10b | 0.22±0.03d | 0.07±0.01c | 0.85±0.22b |

Values show the means of three replicates ± SD. Means followed by same lower case letters are not significantly different at *P≤ 0.05*.
